# 2-Di­chloro­methyl-*N*-ethyl-5-(1-phenyl­silolan-1-yl)cyclo­pent-3-enecarboxamide

**DOI:** 10.1107/S160053681302446X

**Published:** 2013-09-12

**Authors:** Han Xiao, Wan-Qiu Yang, Liang Shen

**Affiliations:** a Chemical Science and Technology Department, Kunming University, Kunming 650091, People’s Republic of China

## Abstract

In the title compound, C_19_H_25_Cl_2_NOSi, the NH group and the carbonyl O atom of the amide fragment are involved in an inter­molecular N—H⋯O hydrogen bond forming chains of mol­ecules. The plane of the benzene ring forms a dihedral angle of 50.5 (2)° with respect to the silolane ring and an angle of 49.74 (2)° with the cyclo­pentyl moiety.

## Related literature

For biological activity of silicon-containing compounds, see: Tacke & Wannagat (1975 ▶, 1979 ▶); Voronkov (1979 ▶). For synthetic methods, see: Matthews *et al.* (2001 ▶, 2002 ▶); Benkeser *et al.* (1962 ▶). For bond-length data, see: Allen *et al.* (1987 ▶).
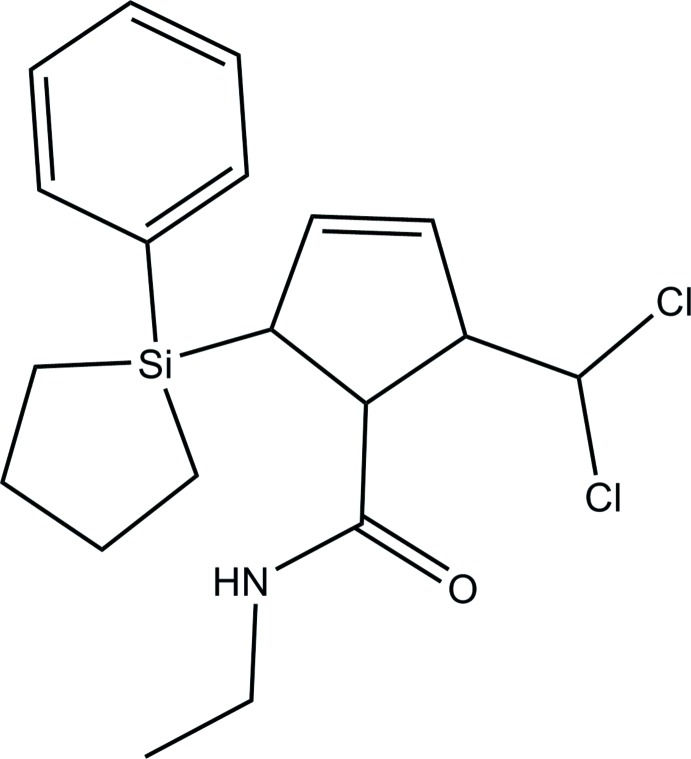


## Experimental

### 

#### Crystal data

C_19_H_25_Cl_2_NOSi
*M* *_r_* = 382.39
Orthorhombic, 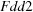

*a* = 42.892 (9) Å
*b* = 13.335 (3) Å
*c* = 14.234 (3) Å
*V* = 8141 (3) Å^3^
*Z* = 16
Mo *K*α radiation
μ = 0.38 mm^−1^
*T* = 295 K
0.20 × 0.10 × 0.10 mm

#### Data collection

Enraf–Nonius CAD-4 diffractometer
Absorption correction: ψ scan (North *et al.*, 1968 ▶) *T*_min_ = 0.927, *T*_max_ = 0.963
3814 measured reflections
1925 independent reflections
1375 reflections with *I* > 2σ(*I*)
*R*_int_ = 0.045
3 standard reflections every 200 reflections intensity decay: 1%

#### Refinement

*R*[*F* ^2^ > 2σ(*F* ^2^)] = 0.048
*wR*(*F* ^2^) = 0.106
*S* = 1.03
1925 reflections
219 parameters
1 restraint
H-atom parameters constrained
Δρ_max_ = 0.17 e Å^−3^
Δρ_min_ = −0.22 e Å^−3^

### 

Data collection: *CAD-4 Software* (Enraf–Nonius, 1985 ▶); cell refinement: *CAD-4 Software*; data reduction: *XCAD4* (Harms & Wocadlo, 1995 ▶); program(s) used to solve structure: *SHELXS97* (Sheldrick, 2008 ▶); program(s) used to refine structure: *SHELXL97* (Sheldrick, 2008 ▶); molecular graphics: *SHELXTL* (Sheldrick, 2008 ▶); software used to prepare material for publication: *publCIF* (Westrip, 2010 ▶).

## Supplementary Material

Crystal structure: contains datablock(s) I, 1R. DOI: 10.1107/S160053681302446X/im2436sup1.cif

Structure factors: contains datablock(s) I. DOI: 10.1107/S160053681302446X/im2436Isup2.hkl

Supplementary material file. DOI: 10.1107/S160053681302446X/im2436Isup3.cml

Additional supplementary materials:  crystallographic information; 3D view; checkCIF report

## Figures and Tables

**Table 1:** Hydrogen-bond geometry (Å, °)

*D*—H⋯*A*	*D*—H	H⋯*A*	*D*⋯*A*	*D*—H⋯*A*
N1—H1⋯O1^i^	0.86	2.10	2.945 (5)	170

Symmetry code: (i) 
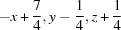
.

**Fig. 1.**
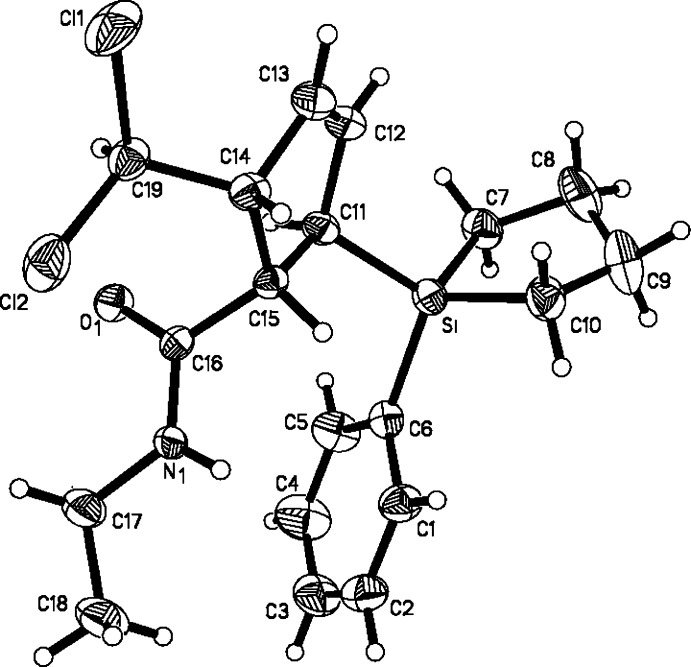
Molecular structure of the title compound with thermal ellipsoids shown at the 30% probability levels).

## supplementary crystallographic information

### 1. Comment

It is well known that some silicon-containing compounds show extremely high biological activity, *e.g.* silatranes, which are much more toxic than strychnine (Tacke & Wannagat, 1975, 1979; Voronkov, 1979). In recent years, people have reported that the reaction of diphenyldichlorosilane with magnesium and butadiene yields silacyclopentenes, which are thought to be formed *via* a diphenylsilylene intermediate (Matthews *et al.*, 2001, 2002). As part of this work, we synthesized the title compound derived from 1-(cyclopenta-2,4-dienyl)-1-phenylsilolane (CDP), and its structure is reported here..

The compound crystallized with a structural configuration in which the phenyl ring (C1~C6) forms a dihedral angle of 50.5 (2)° with respect to the silolane ring (C7,C8,C9,C10,Si1). The cyclopentene ring (C11~C15) is almost planar with the largest deviation being 0.074 Å for atom C15. The bond length of C12—C13 (1.294 (6) Å), agrees with the value characteristic of a double bond. in general bond lengths (Allen *et al.*, 1987) and angles are within normal ranges. In the crystal structure, there one intermolecular hydrogen bond (N1—H1···O1) is observed.

### 2. Experimental

1-(cyclopenta-2,4-dienyl)-1-phenylsilolane (CDP) was synthesized according to the method reported by Benkeser *et al.* (1962). 0.5 mol of CDP was dissolved in 20 ml n-hexane and 20 ml triethylamine in a 200 ml round flask. At 0°C 0.6 mol of 2, 2-dichloroacetyl chloride was added to the flask in 30 min. After continually stirring for 1 h, the solvent was removed and the residue was fractionated on a Todd-column (yield: 31.7%). Colourless block-shaped and needlelike crystals were obtained by slow evaporation of the solution in methanol. Colourless block-shaped single crystals suitable for X-ray structure determination were picked up and determined while the needlelike crystals were too thin to perform an X-ray diffraction experiment. Acoording to elemental analysis, colourless block-shaped and needlelike crystals show an identical composition and are therefore considered to be diastereoisomeric forms of the title compound.

### 3. Refinement

All H atoms were placed in calculated positions, with C—H = 0.93 Å for phenyl, 0.96 Å for methyl, 0.97 Å for methylene and 0.98 Å for methine H atoms, and refined as riding, with *U*_iso_(H) = 1.2 U_eq_(C) for phenyl, methylene and methine H, and 1.5 U_eq_(C) for methyl H atoms.

### Crystal data

**Table:**

C_19_H_25_Cl_2_NOSi	*F*(000) = 3232
*M**_r_* = 382.39	*D*_x_ = 1.248 Mg m^−^^3^
Orthorhombic, *F**d**d*2	Mo *K*α radiation, λ = 0.71073 Å
Hall symbol: F 2 -2d	Cell parameters from 25 reflections
*a* = 42.892 (9) Å	θ = 9–13°
*b* = 13.335 (3) Å	µ = 0.38 mm^−^^1^
*c* = 14.234 (3) Å	*T* = 295 K
*V* = 8141 (3) Å^3^	Block, colourless
*Z* = 16	0.20 × 0.10 × 0.10 mm

### Data collection

**Table:**

Enraf–Nonius CAD-4 diffractometer	1375 reflections with *I* > 2σ(*I*)
Radiation source: fine-focus sealed tube	*R*_int_ = 0.045
Graphite monochromator	θ_max_ = 25.4°, θ_min_ = 1.9°
ω/2θ scans	*h* = −51→51
Absorption correction: ψ scan (North *et al.*, 1968)	*k* = 0→16
*T*_min_ = 0.927, *T*_max_ = 0.963	*l* = −17→0
3814 measured reflections	3 standard reflections every 200 reflections
1925 independent reflections	intensity decay: 1%

### Refinement

**Table:**

Refinement on *F*^2^	Secondary atom site location: difference Fourier map
Least-squares matrix: full	Hydrogen site location: inferred from neighbouring sites
*R*[*F*^2^ > 2σ(*F*^2^)] = 0.048	H-atom parameters constrained
*wR*(*F*^2^) = 0.106	*w* = 1/[σ^2^(*F*_o_^2^) + (0.0512*P*)^2^] where *P* = (*F*_o_^2^ + 2*F*_c_^2^)/3
*S* = 1.03	(Δ/σ)_max_ < 0.001
1925 reflections	Δρ_max_ = 0.17 e Å^−^^3^
219 parameters	Δρ_min_ = −0.22 e Å^−^^3^
1 restraint	Extinction correction: *SHELXTL* (Sheldrick, 2008), Fc^*^=kFc[1+0.001xFc^2^λ^3^/sin(2θ)]^-1/4^
Primary atom site location: structure-invariant direct methods	Extinction coefficient: 0.00184 (15)

### Special details

**Table:**

Geometry. All e.s.d.'s (except the e.s.d. in the dihedral angle between two l.s. planes) are estimated using the full covariance matrix. The cell e.s.d.'s are taken into account individually in the estimation of e.s.d.'s in distances, angles and torsion angles; correlations between e.s.d.'s in cell parameters are only used when they are defined by crystal symmetry. An approximate (isotropic) treatment of cell e.s.d.'s is used for estimating e.s.d.'s involving l.s. planes.
Refinement. Refinement of *F*^2^ against ALL reflections. The weighted *R*-factor *wR* and goodness of fit *S* are based on *F*^2^, conventional *R*-factors *R* are based on *F*, with *F* set to zero for negative *F*^2^. The threshold expression of *F*^2^ > σ(*F*^2^) is used only for calculating *R*-factors(gt) *etc*. and is not relevant to the choice of reflections for refinement. *R*-factors based on *F*^2^ are statistically about twice as large as those based on *F*, and *R*- factors based on ALL data will be even larger.

### Fractional atomic coordinates and isotropic or equivalent isotropic displacement parameters (Å^2^)

**Table:**

	*x*	*y*	*z*	*U*_iso_*/*U*_eq_	
C1	0.94858 (13)	−0.0023 (4)	0.1728 (4)	0.0739 (16)	
H1A	0.9458	−0.0079	0.2374	0.089*	
C2	0.94605 (15)	−0.0875 (5)	0.1173 (5)	0.090 (2)	
H2A	0.9415	−0.1492	0.1446	0.108*	
C3	0.95014 (17)	−0.0805 (6)	0.0235 (6)	0.100 (2)	
H3A	0.9488	−0.1376	−0.0137	0.120*	
C4	0.95610 (19)	0.0076 (7)	−0.0158 (5)	0.116 (3)	
H4A	0.9586	0.0119	−0.0805	0.139*	
C5	0.95859 (15)	0.0936 (5)	0.0391 (4)	0.0888 (19)	
H5A	0.9627	0.1548	0.0103	0.107*	
C6	0.95507 (10)	0.0902 (4)	0.1349 (3)	0.0557 (12)	
C7	0.98950 (12)	0.2923 (4)	0.1792 (4)	0.0792 (17)	
H7A	1.0041	0.2612	0.1360	0.095*	
H7B	0.9818	0.3542	0.1519	0.095*	
C8	1.00467 (18)	0.3112 (6)	0.2756 (6)	0.123 (3)	
H8A	1.0266	0.3264	0.2670	0.148*	
H8B	0.9949	0.3687	0.3051	0.148*	
C9	1.00157 (18)	0.2240 (8)	0.3370 (5)	0.128 (3)	
H9A	1.0062	0.2434	0.4012	0.154*	
H9B	1.0165	0.1731	0.3184	0.154*	
C10	0.96865 (14)	0.1803 (4)	0.3323 (4)	0.0796 (16)	
H10A	0.9549	0.2136	0.3764	0.096*	
H10B	0.9688	0.1089	0.3455	0.096*	
C11	0.91725 (10)	0.2683 (3)	0.2005 (3)	0.0526 (11)	
H11A	0.9139	0.2893	0.1353	0.063*	
C12	0.91344 (13)	0.3580 (4)	0.2632 (4)	0.0716 (15)	
H12A	0.9276	0.4108	0.2644	0.086*	
C13	0.88879 (14)	0.3557 (4)	0.3152 (4)	0.0754 (15)	
H13A	0.8841	0.4044	0.3598	0.090*	
C14	0.86844 (11)	0.2662 (3)	0.2964 (3)	0.0563 (12)	
H14A	0.8647	0.2304	0.3554	0.068*	
C15	0.88968 (9)	0.2007 (3)	0.2312 (3)	0.0448 (10)	
H15A	0.8982	0.1458	0.2692	0.054*	
C16	0.87364 (10)	0.1546 (3)	0.1461 (3)	0.0460 (10)	
C17	0.85258 (15)	0.0022 (4)	0.0751 (4)	0.0873 (18)	
H17A	0.8307	0.0203	0.0759	0.105*	
H17B	0.8611	0.0230	0.0151	0.105*	
C18	0.85540 (18)	−0.1051 (4)	0.0838 (6)	0.113 (2)	
H18A	0.8427	−0.1371	0.0369	0.169*	
H18B	0.8486	−0.1255	0.1451	0.169*	
H18C	0.8768	−0.1243	0.0751	0.169*	
C19	0.83734 (11)	0.2984 (4)	0.2541 (4)	0.0637 (13)	
H19A	0.8411	0.3267	0.1916	0.076*	
Cl1	0.81918 (4)	0.39170 (15)	0.32648 (13)	0.1198 (8)	
Cl2	0.81099 (3)	0.19693 (13)	0.24396 (13)	0.0928 (6)	
N1	0.86865 (8)	0.0559 (2)	0.1503 (3)	0.0522 (10)	
H1	0.8751	0.0231	0.1986	0.063*	
O1	0.86593 (8)	0.2069 (2)	0.0793 (2)	0.0605 (9)	
Si1	0.95663 (3)	0.20524 (10)	0.20897 (9)	0.0551 (4)	

### Atomic displacement parameters (Å^2^)

**Table:**

	*U*^11^	*U*^22^	*U*^33^	*U*^12^	*U*^13^	*U*^23^
C1	0.088 (4)	0.065 (3)	0.070 (4)	0.010 (3)	0.005 (3)	−0.005 (3)
C2	0.105 (5)	0.063 (4)	0.103 (6)	0.012 (3)	−0.001 (4)	−0.011 (4)
C3	0.112 (5)	0.094 (5)	0.093 (6)	0.010 (4)	0.002 (4)	−0.038 (5)
C4	0.167 (7)	0.112 (6)	0.068 (5)	−0.006 (5)	0.024 (5)	−0.023 (4)
C5	0.115 (5)	0.080 (4)	0.071 (4)	−0.016 (4)	0.019 (4)	−0.001 (3)
C6	0.048 (2)	0.069 (3)	0.050 (3)	0.002 (2)	0.009 (2)	−0.005 (2)
C7	0.053 (3)	0.082 (4)	0.102 (5)	−0.016 (3)	0.006 (3)	−0.011 (3)
C8	0.091 (5)	0.145 (6)	0.133 (7)	−0.052 (5)	−0.030 (5)	−0.014 (6)
C9	0.104 (5)	0.190 (9)	0.091 (5)	−0.026 (6)	−0.038 (5)	−0.008 (6)
C10	0.088 (4)	0.091 (4)	0.060 (3)	0.009 (3)	−0.008 (3)	−0.010 (3)
C11	0.061 (3)	0.045 (2)	0.052 (3)	−0.009 (2)	0.001 (2)	0.000 (2)
C12	0.074 (4)	0.049 (3)	0.092 (4)	−0.005 (3)	−0.013 (3)	−0.010 (3)
C13	0.082 (4)	0.072 (3)	0.073 (4)	0.013 (3)	−0.005 (3)	−0.027 (3)
C14	0.071 (3)	0.057 (3)	0.041 (3)	0.008 (2)	0.005 (2)	−0.004 (2)
C15	0.053 (3)	0.043 (2)	0.039 (2)	0.001 (2)	0.000 (2)	0.006 (2)
C16	0.047 (2)	0.046 (3)	0.045 (3)	0.005 (2)	0.0012 (19)	0.006 (2)
C17	0.117 (5)	0.062 (3)	0.083 (4)	−0.009 (3)	−0.034 (4)	−0.007 (3)
C18	0.163 (6)	0.072 (4)	0.104 (5)	−0.024 (4)	−0.038 (5)	−0.016 (4)
C19	0.064 (3)	0.071 (3)	0.056 (3)	0.017 (3)	0.004 (3)	−0.002 (3)
Cl1	0.1200 (14)	0.1454 (16)	0.0939 (12)	0.0787 (12)	0.0007 (11)	−0.0313 (12)
Cl2	0.0573 (8)	0.1037 (11)	0.1173 (13)	0.0021 (8)	0.0057 (8)	0.0253 (10)
N1	0.062 (2)	0.043 (2)	0.051 (2)	−0.0045 (18)	−0.0147 (19)	0.0037 (18)
O1	0.084 (2)	0.0553 (18)	0.0425 (17)	0.0005 (16)	−0.0081 (16)	0.0121 (17)
Si1	0.0507 (7)	0.0603 (8)	0.0541 (7)	−0.0046 (6)	0.0019 (6)	−0.0013 (7)

### Geometric parameters (Å, º)

**Table:**

C1—C6	1.375 (7)	C11—C15	1.550 (6)
C1—C2	1.388 (8)	C11—Si1	1.891 (5)
C1—H1A	0.9300	C11—H11A	0.9800
C2—C3	1.350 (10)	C12—C13	1.291 (7)
C2—H2A	0.9300	C12—H12A	0.9300
C3—C4	1.325 (10)	C13—C14	1.502 (7)
C3—H3A	0.9300	C13—H13A	0.9300
C4—C5	1.393 (9)	C14—C19	1.525 (7)
C4—H4A	0.9300	C14—C15	1.567 (6)
C5—C6	1.372 (8)	C14—H14A	0.9800
C5—H5A	0.9300	C15—C16	1.523 (6)
C6—Si1	1.862 (5)	C15—H15A	0.9800
C7—C8	1.539 (10)	C16—O1	1.224 (5)
C7—Si1	1.875 (5)	C16—N1	1.335 (5)
C7—H7A	0.9700	C17—C18	1.442 (8)
C7—H7B	0.9700	C17—N1	1.461 (6)
C8—C9	1.461 (10)	C17—H17A	0.9700
C8—H8A	0.9700	C17—H17B	0.9700
C8—H8B	0.9700	C18—H18A	0.9600
C9—C10	1.529 (10)	C18—H18B	0.9600
C9—H9A	0.9700	C18—H18C	0.9600
C9—H9B	0.9700	C19—Cl2	1.769 (5)
C10—Si1	1.859 (6)	C19—Cl1	1.794 (5)
C10—H10A	0.9700	C19—H19A	0.9800
C10—H10B	0.9700	N1—H1	0.8600
C11—C12	1.501 (6)		
			
C6—C1—C2	121.8 (6)	C13—C12—C11	114.2 (4)
C6—C1—H1A	119.1	C13—C12—H12A	122.9
C2—C1—H1A	119.1	C11—C12—H12A	122.9
C3—C2—C1	119.8 (7)	C12—C13—C14	113.1 (5)
C3—C2—H2A	120.1	C12—C13—H13A	123.4
C1—C2—H2A	120.1	C14—C13—H13A	123.4
C4—C3—C2	120.2 (7)	C13—C14—C19	110.8 (4)
C4—C3—H3A	119.9	C13—C14—C15	102.2 (4)
C2—C3—H3A	119.9	C19—C14—C15	115.5 (4)
C3—C4—C5	120.6 (7)	C13—C14—H14A	109.3
C3—C4—H4A	119.7	C19—C14—H14A	109.3
C5—C4—H4A	119.7	C15—C14—H14A	109.3
C6—C5—C4	121.5 (6)	C16—C15—C11	110.8 (4)
C6—C5—H5A	119.3	C16—C15—C14	115.7 (4)
C4—C5—H5A	119.3	C11—C15—C14	106.6 (3)
C5—C6—C1	116.2 (5)	C16—C15—H15A	107.8
C5—C6—Si1	122.1 (5)	C11—C15—H15A	107.8
C1—C6—Si1	121.6 (4)	C14—C15—H15A	107.8
C8—C7—Si1	102.6 (4)	O1—C16—N1	123.6 (4)
C8—C7—H7A	111.3	O1—C16—C15	120.7 (4)
Si1—C7—H7A	111.3	N1—C16—C15	115.7 (4)
C8—C7—H7B	111.3	C18—C17—N1	112.6 (5)
Si1—C7—H7B	111.3	C18—C17—H17A	109.1
H7A—C7—H7B	109.2	N1—C17—H17A	109.1
C9—C8—C7	111.4 (5)	C18—C17—H17B	109.1
C9—C8—H8A	109.3	N1—C17—H17B	109.1
C7—C8—H8A	109.3	H17A—C17—H17B	107.8
C9—C8—H8B	109.3	C17—C18—H18A	109.5
C7—C8—H8B	109.3	C17—C18—H18B	109.5
H8A—C8—H8B	108.0	H18A—C18—H18B	109.5
C8—C9—C10	111.2 (6)	C17—C18—H18C	109.5
C8—C9—H9A	109.4	H18A—C18—H18C	109.5
C10—C9—H9A	109.4	H18B—C18—H18C	109.5
C8—C9—H9B	109.4	C14—C19—Cl2	112.1 (3)
C10—C9—H9B	109.4	C14—C19—Cl1	110.4 (3)
H9A—C9—H9B	108.0	Cl2—C19—Cl1	107.5 (3)
C9—C10—Si1	103.3 (5)	C14—C19—H19A	108.9
C9—C10—H10A	111.1	Cl2—C19—H19A	108.9
Si1—C10—H10A	111.1	Cl1—C19—H19A	108.9
C9—C10—H10B	111.1	C16—N1—C17	121.8 (4)
Si1—C10—H10B	111.1	C16—N1—H1	119.1
H10A—C10—H10B	109.1	C17—N1—H1	119.1
C12—C11—C15	102.3 (4)	C10—Si1—C6	113.4 (2)
C12—C11—Si1	114.4 (3)	C10—Si1—C7	96.6 (3)
C15—C11—Si1	113.9 (3)	C6—Si1—C7	114.2 (2)
C12—C11—H11A	108.6	C10—Si1—C11	112.8 (2)
C15—C11—H11A	108.6	C6—Si1—C11	107.3 (2)
Si1—C11—H11A	108.6	C7—Si1—C11	112.4 (2)
			
C6—C1—C2—C3	0.3 (10)	C14—C15—C16—N1	−107.3 (4)
C1—C2—C3—C4	−1.0 (12)	C13—C14—C19—Cl2	−173.0 (4)
C2—C3—C4—C5	0.8 (13)	C15—C14—C19—Cl2	71.5 (5)
C3—C4—C5—C6	0.1 (12)	C13—C14—C19—Cl1	−53.2 (5)
C4—C5—C6—C1	−0.8 (10)	C15—C14—C19—Cl1	−168.8 (3)
C4—C5—C6—Si1	−177.3 (5)	O1—C16—N1—C17	−1.6 (7)
C2—C1—C6—C5	0.6 (9)	C15—C16—N1—C17	178.0 (4)
C2—C1—C6—Si1	177.1 (4)	C18—C17—N1—C16	167.7 (5)
Si1—C7—C8—C9	31.3 (8)	C9—C10—Si1—C6	108.3 (5)
C7—C8—C9—C10	−44.1 (9)	C9—C10—Si1—C7	−11.6 (5)
C8—C9—C10—Si1	32.6 (7)	C9—C10—Si1—C11	−129.4 (5)
C15—C11—C12—C13	−4.7 (6)	C5—C6—Si1—C10	−154.6 (5)
Si1—C11—C12—C13	−128.3 (5)	C1—C6—Si1—C10	29.1 (5)
C11—C12—C13—C14	−3.7 (7)	C5—C6—Si1—C7	−45.2 (6)
C12—C13—C14—C19	−113.5 (5)	C1—C6—Si1—C7	138.5 (4)
C12—C13—C14—C15	10.1 (6)	C5—C6—Si1—C11	80.2 (5)
C12—C11—C15—C16	137.1 (4)	C1—C6—Si1—C11	−96.2 (4)
Si1—C11—C15—C16	−98.9 (4)	C8—C7—Si1—C10	−10.0 (5)
C12—C11—C15—C14	10.5 (4)	C8—C7—Si1—C6	−129.4 (5)
Si1—C11—C15—C14	134.5 (3)	C8—C7—Si1—C11	108.0 (5)
C13—C14—C15—C16	−135.9 (4)	C12—C11—Si1—C10	49.2 (4)
C19—C14—C15—C16	−15.5 (5)	C15—C11—Si1—C10	−68.0 (4)
C13—C14—C15—C11	−12.3 (5)	C12—C11—Si1—C6	174.8 (4)
C19—C14—C15—C11	108.1 (4)	C15—C11—Si1—C6	57.6 (4)
C11—C15—C16—O1	−49.2 (5)	C12—C11—Si1—C7	−58.8 (4)
C14—C15—C16—O1	72.3 (5)	C15—C11—Si1—C7	−176.0 (3)
C11—C15—C16—N1	131.2 (4)		

### Hydrogen-bond geometry (Å, º)

**Table:**

*D*—H···*A*	*D*—H	H···*A*	*D*···*A*	*D*—H···*A*
N1—H1···O1^i^	0.86	2.10	2.945 (5)	170

Symmetry code: (i) −*x*+7/4, *y*−1/4, *z*+1/4.
